# Collaborative care for depression in general practice: study protocol for a randomised controlled trial

**DOI:** 10.1186/s13063-017-2064-7

**Published:** 2017-07-21

**Authors:** Ursula Ødum Brinck-Claussen, Nadja Kehler Curth, Annette Sofie Davidsen, John Hagel Mikkelsen, Marianne Engelbrecht Lau, Merete Lundsteen, Claudio Csillag, Kaj Sparle Christensen, Carsten Hjorthøj, Merete Nordentoft, Lene Falgaard Eplov

**Affiliations:** 1 Mental Health Center Copenhagen, Mental Health Services, Kildegårdsvej 28, DK- 2900 Hellerup, Capital Region of Denmark Denmark; 2 The Research Unit for General Practice and Section of General Practice, University of Copenhagen, Øster Farimagsgade 5, Postboks 2099, 1014 Copenhagen K, Denmark; 3 General Practitioner in Copenhagen, Copenhagen, Denmark; 4 Department of Public Health, Aarhus University, Aarhus, Denmark; 5 Mental Health Center Frederiksberg, Mental Health Services, Nordre Fasanvej 57-59, 2000 Frederiksberg, Capital Region of Denmark Denmark; 6 Stolpegård Psychotherapy Center, Mental Health Services, Stolpegårdsvej 20, 2820 Gentofte, Capital Region of Denmark Denmark; 7 Mental Health Center North Zealand, Mental Health Services, Dyrehavevej 48, 3400 Hillerød, Capital Region of Denmark Denmark; 8 Research Unit for General Practice, Institute of Public Health, Aarhus University, Bartholins Allé 2, 8000 Aarhus C, Denmark; 9 Institute for Clinical Medicine, University of Copenhagen, Mental Health Center Copenhagen, Mental Health Services, Kildegårdsvej 28, DK-2900 Hellerup, Capital Region of Denmark Denmark

**Keywords:** Mood disorders, Depression, Collaborative care, Shared care, Detection of depression, General practice, Illness recognition, Cluster randomised trial

## Abstract

**Background:**

Depression is a common illness with great human costs and a significant burden on the public economy. Previous studies have indicated that collaborative care (CC) has a positive effect on symptoms when provided to people with depression, but CC has not yet been applied in a Danish context. We therefore developed a model for CC (the Collabri model) to treat people with depression in general practice in Denmark. Since systematic identification of patients is an “active ingredient” in CC and some literature suggests case finding as the best alternative to standard detection, the two detection methods are examined as part of the study. The aim is to investigate if treatment according to the Collabri model has an effect on depression symptoms when provided to people with depression in general practice in Denmark, and to examine if case finding is a better method to detect depression in general practice than standard detection.

**Methods/Design:**

The trial is a cluster-randomised, clinical superiority trial investigating the effect of treatment according to the Collabri model for CC, compared to treatment as usual for 480 participants diagnosed with depression in general practice in the Capital Region of Denmark. The primary outcome is depression symptoms (Beck’s Depression Inventory (BDI-II)) after 6 months. Secondary outcomes include depression symptoms (BDI-II) after 15 months, anxiety symptoms (Beck’s Anxiety Inventory (BAI)), level of functioning (Global Assessment of Function (GAF)) and psychological stress (Symptom Checklist-90-Revised (SCL-90-R)). In addition, case finding (with the recommended screening tool Major Depression Inventory (MDI)) and standard detection of depression is examined in a cluster-randomized controlled design. Here, the primary outcome is the positive predictive value of referral diagnosis.

**Discussion:**

If the Collabri model is shown to be superior to treatment as usual, the study will contribute with important knowledge on how to improve treatment of depression in general practice, with major benefit to patients and society. If case finding is shown to be superior to standard detection, it will be recommended as the detection method in future treatment according to the Collabri model.

**Trial registration:**

ClinicalTrials.gov. NCT02678845. Retrospectively registered on 7 February 2016.

**Electronic supplementary material:**

The online version of this article (doi:10.1186/s13063-017-2064-7) contains supplementary material, which is available to authorized users.

## Background

### The treatment outcome study

Depression is a common disorder with a lifetime risk of 17–18% [1]. A Danish survey found a point prevalence of major depression of 3.3% [2]. Based on the Danish National Patient Register [3], including information on all patients in Danish hospitals, the annual number of new cases of depression admitted to a psychiatric hospital is 11,000. It is estimated that 32,000 men and 59,000 women are living with depression [4]. The human cost relating to depression is great, but the illness also places a significant burden on the public economy. It is estimated that the cost related to loss of production due to depression in Denmark is approximately 0.5 billion US dollars per annum [4]. The majority of people with depression are treated in general practice [5], but studies show that many patients with depression go unrecognised or do not receive evidence-based treatment in general practice [2, 5–7]. Some of the obstacles in the current management and organisation of treatment of depression that have been identified are as follows. First, there is a lack of coordination of the management of depression, as there is no organised, coherent treatment regime between general practice and psychiatry. Second, there is a lack of treatment opportunities, as most general practitioners (GPs) are not trained to offer psychotherapy. It is estimated that only around one third of GPs in Denmark are qualified to offer cognitive behavioural therapy (CBT) [6]. However, GPs can refer patients with mild and moderate depression to a psychologist, but part of the treatment is paid for by the patient (with the other part being publically funded if the patient meets specific criteria). Third, there is a shortage of independent psychiatrists and psychologists trained in CBT or other evidence-based psychotherapies targeting depression, delaying the specialised treatment of patients referred from general practice [6].

National guidelines recommend that optimising depression treatment in general practice could be done through introducing shared-care interventions, such as collaborative care (CC) programmes [1]. CC stems from the recognition, that patients with depression in general practice may profit from an organisation of care corresponding to the model introduced for other chronic diseases [7]. Health economics studies indicate that, in spite of extra initial costs when introducing CC, the costs tend to have recovered after 3–4 years and with substantial long-term savings, because of reduction in sick leave and disability pensions [8].

A Cochrane review from 2012, investigating the effects of CC for depression, concluded that CC is associated with significant improvements in treatment outcomes for up to 2 years, compared with usual care [9]. Therefore, CC represents a useful addition to clinical pathways for adult patients with depression. The evidence is based on research primarily conducted in the USA and England. Until now, no studies have been conducted in Scandinavia. The current evidence is therefore based on an organisational framework not directly applicable in a Danish context, and it is not possible to know whether CC will have the same effect in a Danish context [10]. Thus, it is necessary to adjust the model to Danish conditions. The development of a Danish model for CC (the Collabri model) for depression was completed in 2014 in cooperation between Danish GPs, psychiatrists and researchers. A protocol for a cluster-randomised trial in patients with depression treated according to the Collabri model, are presented in this article. At the same time models for CC for panic disorder, generalized anxiety and social phobia were developed. The trial protocol for the investigation of CC vs. treatment as usual for these three anxiety disorders are presented in a separate paper.

### The detection of depression study

Bower et al. reports that systematic identification of patients is an “active ingredient” in CC [11], so we want to include systematic identification in the Collabri depression study, and to use a method that can be carried out by GPs. Studies indicate that GPs fail to diagnose about half of patients with depression in general practice [12, 13]. There is substantial evidence that routine screening will lead to too many false positives [12], because of the modest prevalence of depression in primary care. However, the US Preventive Task Force reports evidence supporting screening for depression [14]. One factor for the opposing conclusions is that the latter includes research where screening is supported by subsequent treatment [15].

To sum up, screening for depression is recommended in the USA, but not in the UK and in Denmark, where screening is only recommended in high-risk groups [1]. Nevertheless, screening of high-risk groups is not supported by subsequent literature. A randomised controlled trial on high-risk screening found no difference in recognition rates between the intervention group and the control group [16]. In a prospective cohort study, as a final result of screening, only 1% (17/1687) started treatment for major depressive disorder [17]. Finally, in a Danish observational study investigating high-risk screening for depression with the Major Depression Inventory (MDI), compared with screening with MDI on clinical suspicion (called case-finding) and a combination of the two methods, investigators found that screening of patients in high-risk groups had limited effect in addition to the cheaper and less invasive method of case-finding [18].

In the reviewed randomized controlled trials (RCTs), screening was conducted using a variety of other tools including the General Health Questionnaire (GHQ), Patient Health Questionnaire (PHQ)-9, Primary Care Evaluation of Mental Disorders (PRIME-MD), and hospital anxiety and depression scale (HADS), but we planned to use the MDI, a self-assessment questionnaire that uses the diagnostic criteria from *International Classification of Diseases edition 10* (ICD-10) [19] and is recommended for screening and diagnostics of depression in Denmark [1]. It has shown good agreement with standard methods such as Schedules for Clinical Assessment in Neuropsychiatry (SCAN), [20] for both diagnosis and severity [1, 21]. Based on the aforementioned literature and the available techniques, case-finding is tested against standard detection of depression in a randomised controlled design as part of the Collabri study. Since the study on detection of depression (the detection study) is incorporated with the Collabri studies and shares much of their methods, this article presents both the protocol (version 2) for a cluster randomised trial on treatment according to the Collabri model for patients with depression (the treatment outcome study) and for the study on detection of depression (the detection of depression study).

## Methods

### Aim and design

The aim of the study is to test the null hypothesis that treatment according to the Collabri model (intervention group) and treatment as usual (control group) has the same effect on depression symptoms in people with depression in primary care and to test the null hypothesis that the performance of case-finding and standard detection of depression in primary care is the same. The treatment outcome study is a cluster-randomised, clinical superiority trial in 480 patients diagnosed with depression in general practice. The detection of depression study is a cluster-randomised trial within the Collabri study setting.

### Eligibility of study participants

#### Cluster level

GPs with a registered provider number in the Capital Region of Denmark, except for Bornholm, are eligible to participate in the Collabri study of depression. The local branch of the Organisation of General Practitioners in Denmark and the Capital Region of Health Care have negotiated and signed an agreement that allows the GPs to participate in the study and sets out the terms and conditions for it, including financial reimbursement.

#### Individual level

Patients are eligible for the outcome study and the detection of depression study if their GP participates in the study and the patient complies with the following inclusion and exclusion criteria, assessed either by the GP at recruitment and/or by a research assistant at a baseline eligibility interview. Patients are eligible for the outcome study if they are diagnosed with depression according to the ICD-10. Patients are eligible for the detection of depression study if they are referred to the study either by their GP in the standard detection group with a depression diagnosis or by their GP in the case-finding group with a (positive or negative) MDI score. In both studies, patients must be 18 years of age, Danish speaking and give her/his written informed consent to participate in the trial on the terms described.

Patients cannot participate in the Collabri studies and are excluded at baseline if they have a high risk of suicide, a current psychotic condition, obsessive compulsory disorder (OCD), post-traumatic stress disorder (PTSD), bipolar affective disorder, or alcohol or substance misuse that prevents the person from participating in the Collabri intervention. Patients are also excluded at baseline if they are in current therapeutic or medical treatment for anxiety or depression, have a pending disability pension case, or have been treated for anxiety or depression within the last 6 months. Additionally, patients diagnosed with dementia and patients assessed by the GP to be medically unstable, making it impossible to adhere to treatment, cannot participate. Further, patients cannot participate if they are referred to treatment in the secondary psychiatric caresystem not later than at first contact with the GP after inclusion by a research assistant is referred to treatment in the secondary psychiatric care system. Patients in the intervention group are excluded if they want treatment cf. the psychologist scheme and do not want referral to the psychologist to be preceded by treatment according to the Collabri model.

### Recruitment and randomisation

#### Cluster level

Recruitment of GPs ran from May 2014 to July 2015. GPs in the catchment areas of the Capital Region in Denmark were invited to join the study through letters with information about the project and dates for a total of four information meetings. Additionally, the GPs were recruited through articles in professional newsletters and forums. Information visits and telephone calls to GPs were made on request.

**Fig. 1:**
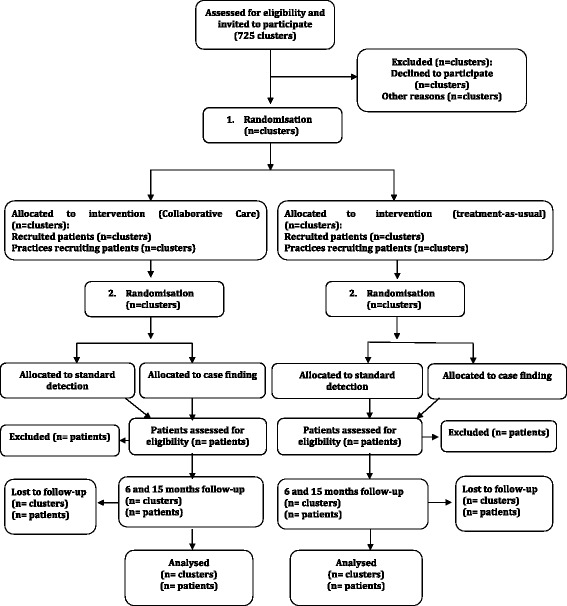
Flow-chart for participants

The randomisation was conducted by centralised random computer-generated allocation sequence, carried out externally by the Research Centre for Prevention and Health (RCPH) at practice level, where each cluster corresponds to a provider number consisting of one or more GPs. Cluster randomisation was chosen because of the risk of bias in the form of contamination if the randomisation was on an individual level. The GPs were randomised to give either treatment as usual or CC according to the Collabri model. In both the treatment as usual group and the CC group, the GPs were subsequently randomised according to detection method, which was either standard detection of depression or case-finding. In standard detection the GPs were asked to use the MDI [19] as often as they usually did. In case-finding the GPs were asked to systematically use the MDI as a screening tool if they suspected depression. See Fig. 1 for an illustration of the randomisation process.

#### Individual level

Patients within the clusters are allocated to the same group as their GP. The GPs enrolled in the study detect patients with depression according to randomisation allocation. Only patients who meet the criteria for depression at baseline are included in the depression study. Patients who have not initially been diagnosed with depression by the GP and have only been referred to the detection of depression study, and are subsequently diagnosed with depression through the Mini International Neuropsychiatric Interview (MINI), are asked to participate in the treatment outcome study if the GP agrees with the diagnosis.

The GP obtains verbal consent from the patient that a research assistant can make contact by telephone in order to conduct a diagnostic interview (MINI interview). If the diagnosis according to the MINI interview is inconsistent with the GP’s diagnosis, the research assistant consults a psychiatrist in the Collabri group, who contacts the GP to confirm which diagnosis is correct. Patient recruitment was initiated in November 2014 and ended on 31 December 2016.

### Blinding

It is not possible to ensure blinding of the patient and GP, but the blinding of GP allocation to intervention group is maintained for researchers in the data collection phase and in the analysis phase. The intervention groups are coded and anonymised (e.g. X and Y) so that researchers are blinded in the entire phase of the analysis and when writing the conclusion. In the detection of depression study, blinding of the patient diagnosis and the MDI result is maintained for the researchers during the MINI interview, in order to ensure that the evaluation of diagnosis is not affected by the GP’s referral diagnosis.

Care managers, GPs, psychiatrists, and patients are notified that they cannot reveal to the researchers to which group the patients are allocated. If the blinding is broken and referral diagnosis, or baseline or outcome measures are revealed at the baseline interview or at the follow-up interview, the patient is referred to another blinded researcher.

### The experimental intervention - treatment according to the Collabri model

Four criteria for CC are listed in the Cochrane review [9]: a multi-professional approach to treatment, scheduled monitoring and review, enhanced inter-professional communication, and a structured treatment plan. Participants in the intervention group are treated according to the Collabri model, based on the four criteria listed in the Cochrane review [9] and on the recommendations from a systematic literature reveiw by Eplov et al. [8], and adapted to the Danish health care system by including collaboration with relevant social worker(s) in the municipality and integrating the existing psychologist scheme. On the basis of these and on current guidelines [1, 22–25], the following elements were integrated into the Collabri model: recruitment of staff with mental health care experience, training of GP and care manager, use of instruments for detection and follow up, education and treatment of the patient, supervision from a psychiatric specialist, and a stepped-care approach to treatment where treatment is always commenced on the least invasive and least resource-demanding level. These elements will be described in detail.

The Collabri model is based on a multi-professional approach to treatment. Treatment is provided by care managers, GPs and psychiatrists, all of whom receive a short training following their recruitment. The care manager has a medium-long health professional education, is often a nurse, and has experience in working in mental health together with a certified CBT course (of minimum 1 year). The care manager provides assessment of side effects due to medical treatment, CBT, supportive conversations, diagnosis-specific treatment, psychoeducation and regular contact with social worker(s). The GP has overall responsibility for treatment and takes care of the diagnostic procedure, initiation of treatment, coordination of the treatment intervention with the care manager, supervision of the care manager, and collaboration with the care manager on contact with social caseworker(s). The psychiatrist guides and supervises the care manager and GP and participates in joint consultations with the GP.

According to the Collabri model, the patient is regularly monitored and assessed at scheduled patient follow-up visits. Monitoring consists of assessments at intervals determined by the severity of depression or anxiety - at least every 2 weeks, more frequently on signs of significant change in the condition and at least once per week when stepping up medication. Review will take place at least once a month and always at the end of a treatment component.

The Collabri model includes enhanced inter-professional communication where the care manager and the GP meet at minimum weekly and discuss patients. In addition, the psychiatrist supervises the care managers in groups twice a month, and the GPs in groups once a month. Furthermore, the psychiatrist supervises the care managers and GPs individually when needed.

The psychiatrist, GP, and care manager may have joint consultations if needed. Communication between professionals can take place via video conferences if it is not possible to meet in person; however, the weekly communication between the care manager and GP must be face to face.

The CC treatment for depression and anxiety takes place according to treatment guidelines established on the basis of the Danish Health Authority’s reference programme for unipolar depression in adults [1], The Danish College of General Practitioners’ guideline for unipolar depression [22], the Danish Health Authority’s reference programme for anxiety disorders [24] and The Danish College of General Practitioners’ clinical guideline for anxiety disorders [25].

The Collabri model also focuses on the principles of a stepped-care approach to treatment, active and planned follow up, patient involvement and influence on treatment, involvement of carers, and self-management.

The specific treatment modalities in the Collabri model are medical treatment in accordance with treatment plans in Collabri, psychotherapy in the form of CBT by a care manager in accordance with treatment plans in Collabri, psychoeducation in groups in the form of the course “Lær at takle angst og depression” (“Learn to cope with anxiety and depression”), based on the Chronic Disease Management Program (http://patienteducation.stanford.edu/programs/cdsmp.html) or individual psychoeducation carried out by the care manager or psychoeducation as a part of CBT. All patients and carers are offered written psychoeducational material.

### Fidelity

To ensure the internal validity of the intervention, an evaluation (fidelity measurement) is carried out after 6 months and at least once more during the project period. The fidelity measurement ensures that the intervention is carried out according to the described Collabri model. Based on the assessments, an action plan is developed if needed in order to improve the implementation. The fidelity scale is available through the corresponding author.

### The control intervention - treatment as usual group

**Fig. 2:**
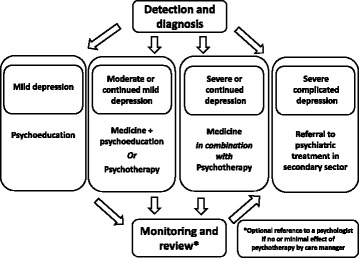
Stepped-care plan

GPs in the treatment as usual group treat the participating patients as they normally do. The treatment is based on clinical guidelines from The National Board of Health and The Danish College of General Practice, including recommendations on detection, diagnosis, treatment, and referral [23]. The guidelines recommend screening for depression in risk groups, use of screening tools, e.g. the World Health Organization (WHO)-5 Well-being Index (WHO-5) or use of two screening questions, diagnosis according to the ICD-10, and treatment according to a stepped-care model with the following treatment elements: psychotherapy (for mild to moderate depression) and medical treatment (for moderate depression not responding to therapy and for severe depression) (see Fig. 2). The guidelines also recommend, in some cases, admission to an inpatient ward or referral to or advice from a psychiatrist, community mental health centre, or a psychologist.

### Assessments and outcome measures

#### Baseline eligibility interview

The GP assesses referral diagnosis and comorbidity at recruitment and refers the participant to the study. At baseline, the participants are interviewed by telephone in order to assess the inclusion and exclusion criteria and confirm the diagnosis on the basis of a modified version of the MINI [26], including ICD-10-specific questions for the inclusion diagnosis.

#### Outcome measures in the treatment outcome study

The primary outcome for the treatment outcome study is self-reported degree of depression measured by the Beck Depression Inventory (BDI-II) [27] at 6 months. BDI-II is a 21-item multiple-choice questionnaire assessing degree of depression and changes in depression mode by measuring symptoms of depression during the last 14 days. Each item has four statements reflecting increase in symptom severity from scores 0 to 3. The total score ranges from 0 to 63.

Secondary outcomes for the treatment outcome study are self-reported degree of depression measured by the BDI-II [27] at 15 months, self-reported degree of anxiety measured by the Beck Anxiety Inventory (BAI) [28] at 6 months, functional impairment measured with the Global Assessment of Functioning (GAF-F, split version) [29] obtained through a semi-structured interview with a research assistant, and self-reported psychological stress measured with the Symptom Checklist (SCL-90-R) [30] at 6 and 15 months.

The BAI is a 21-item multiple-choice questionnaire assessing physiological anxiety symptoms. Each item has four statements scoring 0 to 3, reflecting increase in the degree to which the participant has been bothered with the symptom over the last week. The total score ranges from 0 to 63. The GAF-F measures the participants’ level of functioning over the past month on a continuous scale ranging from 0 to 100. The SCL-90-R is a multi-dimensional questionnaire assessing mental health problems and psychopathological symptoms on a continuous scale from the lowest functional level of 0 to the highest functional level of 100. It consists of 90 items, each item graded on a scale of 0–4, based on the extent to which the participant has experienced the symptom within the past week. The questionnaire measures nine primary dimensions of symptoms and can be summed in the global severity index (GSI), measuring overall psychological distress.

Explorative outcomes for the treatment outcome study are self-reported degree of anxiety measured by the BAI [28] at 15 months, self-reported quality of life measured with the WHO-5 [31], psychosocial functioning measured with the Personal and Social Performance Scale (PSP) [32], obtained through semi-structured interview with a research assistant, self-reported side effects measured by the Patient-Rated Inventory of Side Effects (PRISE) [33], self-reported health-related quality of life measured with the EuroQol Five Dimensions Questionnaire with Three Levels (EQ-5D-3 L) [34], self-reported functional impairment measured with the Sheehan Disability Scale (SDS) [35], self-reported self-efficacy measured with the Illness Perception Questionnaire Revised (IPQ-R) [36] and two subscale questions from the Chronic Disease Self-efficacy Scales (SECD-32) [37], self-reported recovery measured with INSPIRE [38], self-reported general satisfaction with the treatment measured with the Client Satisfaction Questionnaire-8 (CSQ-8) [39], and apathia measured by a research assistant with the Diagnostic Apathia Scale [40], a 6-item rating scale covering neuropsychological symptoms. The number of sick leave days, and information on employment and the use of social services will be obtained from the Danish Register for Evaluation of Marginalization (DREAM) database [41]. The DREAM database contains information on all Danish transfer payments on a weekly basis. Medication use will be obtained from the Danish National Prescription Registry [42], containing detailed information on prescriptions dispensed in Denmark. Demographic data will be obtained from Statistics Denmark [43], covering statistics on Danish society, and the National Prescription Registry [42].

The WHO-5 is a 5-item questionnaire measuring mental health within the last 2 weeks. Each item is rated on a Likert scale from 0 (not present) to 5 (constantly present). The score from 0 to 25 is transformed into a scale from 0 to 100, where higher scores mean better well-being. The PSP is a 100-item scale measuring personal and social functional level within the last month. The score is based on the assessment of performance within 4 domains: (1) socially useful activities, (2) personal and social relationships, (3) self-care, and (4) disturbing and aggressive behaviours. Higher scores mean better functional level. PRISE is a questionnaire used to identify and evaluate the tolerability of side effects within 9 domains. The EQ-5D-3 L measures health status within 5 domains: (1) mobility, (2) self-care, (3) usual activities, (4) pain and discomfort, and (5) anxiety and depression. The participants self-rate their level of severity on a 3-level scale and mark their health status on a visual analogue scale from 0 (worst imaginable health status) to 100 (best imaginable health status). The SDS is a self-report tool measuring functional impairment in 3 domains: (1) work and school, (2) social life, and (3) family life. Symptoms are rated on a visual analogue scale and can be translated into a single dimensional measure of global functional impairment, ranging from 0 (unimpaired) to 30 (highly impaired). The subscales from the SECD-32 measure self-efficacy. The participants rate how confident they are in doing certain activities. Each item is rated on a 10-point Likert scale from 1 (not at all confident) to 10 (very confident). The INSPIRE questionnaire assesses the participant’s feeling of being supported in their recovery by their health care provider (the GP and possibly the care manager). The first 20-item part covers support from the therapist and the second 7-item part covers the relationship with the therapist. The IPQ-R subscale on personal control measures confidence in one’s own ability to influence disease. Each of the 6 items is self-rated on a 5-point Likert scale from 1 (very disagree) to 5 (strongly agree). The CSQ-8 is an 8-item questionnaire that measures general satisfaction with the treatment. Each item is rated on a scale from 0 to 4.

At baseline the following will also be registered: somatic comorbidity reported by the GP at the point of referral, and personality disorder measured by the Standardized Assessment of Personality: Abbreviated Scale (SAPAS) [44], an 8-item screening interview.

#### Safety measures

Self-reported anxiety and depression symptoms are measured by the BAI and BDI-II. Suicidal ideation is evaluated in the MINI interview. Data on death are obtained from the Danish Cause of Death Register [45]. The number of outpatient services, admissions, inpatient days, and life-threatening conditions for reasons other than suicide attempts are obtained from the Danish National Patient Registry (Landspatientregistret) [3] containing data on Danish patients’ contacts with hospitals in Denmark. Numbers of sick leave days are obtained from the DREAM database.

#### Outcome measures in the detection of depression study

The primary outcome for the detection of depression study is the positive predictive value of referral diagnosis in two randomised detection settings: case-finding and standard detection. In the standard detection group, based on results from the referral diagnosis performed by the GP and the MINI carried out by the research assistant, the positive predictive value of the GP’s diagnosis is calculated. In the case-finding group, based on results from the MDI and MINI, the positive predictive value of the diagnosis and the sensitivity, specificit, and negative predictive value of the diagnosis are calculated.

### Data collection

The data for the outcome study will consist of interviewer-based data (telephone interview), register data, and self-reported data. Participants will be interviewed by telephone at baseline, and again after 6 and 15 months in order to collect data for GAF and PSP. SAPAS is collected at the baseline interview. Data collection through interviews will be conducted by staff trained in the specific instruments. The participants also complete self-assessment questionnaires at baseline, and again after 6 and 15 months. Self-assessments will be completed electronically, but a paper version can be posted if preferred. If one or more items in the questionnaire have not been answered, the patient will be contacted by the research assistant in order to complete the questionnaire and to clear any doubts the patient may have. Services in relation to the Collabri intervention will be registered by the care managers and psychiatrists. See Table 1 for schedule of enrolment, interventions and assessments.

**Table 1:** Schedule of enrolment, interventions and assessments

Study period							
		Recruitment	Baseline	Post-baseline			Source of data collection
	Timepoint			Treatment period	Follow up, 6 months	Follow up, 15 months	
Enrolment	Referral diagnosis, information and informed consent	x					GP
	Assessment of somatic comorbidity	x					GP
	Eligibility interview (including MINI)		x				Interview
	Standardized Assessment of Personality, Abbreviated Scale		x				Interview
	Suicidal ideation		x				Interview
Interventions	Collaborative care			x			
	Treatment as usual			x			
Assessments	Beck Depression Inventory-II		x		x	x	Self-report
	Beck Anxiety Inventory		x		x	x	Self-report
	Global Assessment of Functioning		x		x	x	Self-report
	Symptom Checklist-90-Revised		x		x	x	Interview
	World Health Organization-5		x		x	x	Self-report
	Personal and Social Performance Scale		x		x	x	Interview
	Patient-Rated Inventory of Side Effects		x		x	x	Self-report
	EuroQol Five-Dimension Questionnaire with Three Levels		x		x	x	Self-report
	Sheehan Disability Scale		x		x	x	Self-report
	Subscale from Illness Perception Questionnaire Revised scale		x		x	x	Self-report
	Chronic Disease Self-efficacy Scales-32		x		x	x	Self-report
	INSPIRE				x		Self-report
	Client Satisfaction Questionnaire-8 + project-specific questions				x		Self-report
	The Diagnostic Apathia Scale		x		x	x	Interview
	Sick leave		x		x	x	Register
	Medication use		x		x	x	Register
	Death		x		x	x	Register
	Life-threatening conditions		x		x	x	Register
	Outpatient services, admissions and inpatient days		x		x	x	Register
	Use of social services		x		x	x	Register
	Intervention-specific services and treatment			x			Care managers and psychiatrists

***MINI*** Mini International Neuropsychiatric Interview

### Training and inter-rater reliability

All assessors have received the necessary training in the relevant instruments and receive ongoing support and supervision. All assessors have participated in regular joint ratings for PSP and GAF, in order to ensure inter-rater reliability. All cases of discrepancy between the referral diagnosis and a diagnosis made at the inclusion interview via the MINI interview will be discussed with the psychiatrist and the referring GP.

### Power and sample size

We made two sample size calculations, one for the depression treatment outcome study and one for the detection of depression study to see how many people to include in the depression treatment outcome study with the embedded detection of depression study

#### Sample-size calculation for the depression treatment outcome study

The primary outcome is a change in the BDI-II summary score. Clinical relevant treatment response at group level is defined as a difference in the degree of depression by 4 points measured by the BDI-II [46, 47]. No surveys have been carried out in Denmark that can contribute to the estimation of the standard deviation (SD) for BDI-II. However, according to international surveys, the SD for BDI-II can be set at 11 [46, 48–50]. There is no knowledge of the size of the intra-class correlation (ICC) in a Danish context; however a review of ICCs in depression in primary care suggests that the ICC can be set at 0.04 [51].

The sample-size calculation for the depression study based on the aforementioned figures shows that 328 participants with depression should be included in order to be able to reject the null hypothesis in the depression study. According to the null hypothesis, participants in the intervention group and the control group improve similarly in terms of symptoms with a power of 0.8 and a significance level of 0.05.

#### Sample size calculation for the detection of depression study

On the basis of a study carried out by Michell et al. [13], the positive predictive value of standard detection is estimated to be 45%. A clinical meaningful and possible [52] increase in the number is estimated to be up to detection of 60%. With the detection of depression study embedded in the depression study, the sample size calculation shows that 480 individuals should be included in order to be able to reject the null hypothesis in the depression study. According to the null hypothesis, participants in the intervention group and the control group improve similarly in terms of symptoms, with a power of 0.8 and a significance level of 0.05.

#### Summary

In all, 480 individuals should be included in the Collabri treatment for depression outcome study with the embedded detection of depression study in order to reject the null hypothesis.

#### Secondary outcomes

The power for the secondary outcomes for depression is estimated to be over 0.8 for all analyses [53–55]. See Table 2.

**Table 2:** Power calculation for the secondary outcomes

Secondary outcome	Mean difference (MD)	Standard deviation (SD) of the pooled mean	Type 1 error	Calculated power
BAI	4 (57)	12 (57)	5%	99%
GAF-F	5 (*)	10 (56)	5%	99%
SCL-90-R	23 (55)	50 (55)	5%	99%

*Abbreviations*: *MD* mean difference, *SD* standard deviation, *BAI* Beck Anxiety Inventory, *GAF-F* Global Assessment of Functioning, *SCL-90-R* Symptom Checklist-90-Revised. *The expected mean difference for GAF-F has been conservatively estimated to 5 points as this is considered clinically relevant

### Feasibility

By including 48 GPs, the GPs must each include 10-11 people with depression in the depression treatment outcome study with the embedded detection of depression study over a 15-month period. This is possible as 12 months prevalence rates indicate that a GP with 1600 registered patients on average will see 140 patients with depression per year [2].

Eight full-time care managers and one and a half psychiatrists (one full-time and one part-time) are employed in the Collabri project. Each care manager has a maximum caseload of 100 participants per year. Thus, it is realistic in terms of the capacity of care managers to complete the intervention in 240 participants with depression.

### Statistical analysis

The Collabri study will be conducted according to the statistical principle “intention-to-treat” [56], which means that once a person is included in the project, he or she stays in the study population and is followed, regardless of whether the person is later excluded. In the detection of depression study the positive predictive value will be calculated and compared for both groups. Subgroup analysis will be performed in patients with a medical comorbidity and a personality disorder.

Linear mixed-effects regression models will be used to compensate for the cluster randomisation and potential confounders. To account for repeated measures, multilevel regression models with random effects will be used, estimated using an unstructured covariance matrix if possible. If not possible, other covariance matrices such as independent, interchangeable, auto-regressive, and Toeplitz will be estimated, and the best-fitting structure selected based on Bayes’ information criterion. This makes it possible to take “missing at follow up” into account under the assumption of “missing at random” by including covariates that are associated with missing values at follow up. The analysis levels are by GP, patients, and time. This method introduces less bias than the method whereby the last observation is used instead of the missing values, and the method whereby only cases with complete follow-up information are eligible for the analysis.

### Project organisation

The project is led by a steering group. The aim of the steering group is to ensure the progress of the research project. A lead project manager ensures the general management of the project together with a project manager in charge of implementation of the intervention and a project manager in charge of the research project. Administrative staff support the project managers. Data collection and analysis are carried out by Ph.D. students and research assistants.

## Discussion

The Collabri model is based on the evidence supporting CC in other countries. To our knowledge this is the first randomised trial investigating the effect of a CC model for depression in a Scandinavian context. The strength of the outcome study is the centralised computer-based cluster randomization, which ensures adequate generation of the allocation sequence and adequate allocation concealment. The use of blinded outcome assessors for the secondary outcome, the fact that it is a register-based outcome, and the use of intention-to-treat analysis decreases the risk of biased effect estimates. The strength of the detection of depression study is also the centralised computer-based cluster randomisation and the use of blinded assessors of the outcomes. The trial is registered at http://www.clinicaltrials.gov, which helps prevent selective and incomplete outcome reporting. The fact that we monitor fidelity to the Collabri model after 6 months is also a strength of the trial. This is intended to ensure that care managers, psychiatrists, and GPs are true to the model. A limitation of this trial is that we are not able to blind participants, care managers, psychiatrists, or GPs to the allocation. Another limitation is that the primary outcome is self-reported and therefore not blinded, which may well lead to overestimation of treatment effects. Some might argue that it is difficult to sustain the blinding of the assessor during follow up, and this certainly leads to risk of bias. Should blinding be broken, a second assessor will complete the follow-up interview.

Although participants are recruited from general practices throughout the Capital Region of Denmark, and should be fairly representative of the population in the region, there may be reduced external validity. As it is the GP who identifies eligible participants, not everybody with depression who is eligible may be asked to participate, as patients are not systematically screened for eligibility. Due to differences in healthcare systems, the results may not be directly comparable with other countries, but together with other trials in the area, it will give a more complete picture of the effects of collaborative care in people with depression.

The results of this trial will add to the knowledge of collaborative care for depression in a Scandinavian context. If the Collabri model is shown to be more effective than treatment as usual in general practice, the results can contribute to improving the care and treatment for patients with depression in general practice with benefits to both patients and society. The results of the detection of depression study add to the knowledge of detection of depression in primary care. If case-finding is shown to be superior to standard detection, it will be recommended as a detection method in future treatment according to the Collabri model.

### Trial status

Recruitment of participants within the clusters is ongoing and continued until the 31 December 2016.

## Additional file

Additional file 1: SPIRIT 2013 checklist: recommended items to address in a clinical trial protocol and related documents*. (DOC 124 kb)

## Abbreviations

BAI: Beck Anxiety Inventory
BDI-II: Beck Depression Inventory
CBT: Cognitive behavioural therapy
CC: Collaborative care
CSQ-8: Client Satisfaction Questionnaire
DREAM: Danish Register for Evaluation of Marginalisation
EQ-5D-3L: EuroQol Five Dimensions Questionnaire with Three Levels
GAF-F: Global Assessment of Functioning
GP: General practitioner
HADS: Hospital anxiety and depression scale
ICC: intra-class correlation
IPQ-R: Illness Perception Questionnaire Revised
MINI: Mini International Neuropsychiatric Interview
PRISE: Patient Rated Inventory of Side Effects
PSP: Personal and Social Performance Scale
RCT: Randomised controlled trial
SAPAS: Standardized Assessment of Personality, Abbreviated Scale
SCL-90-R: Symptom Checklist-90-Revised
SDS: Sheehan Disability Scale
SECD-32: Chronic Disease Self-efficacy Scales
SPS: Personal and Social Performance Scale
WHO-5: World Health Organization-5 Well-being Index
